# Challenges and Opportunities of US and Arab Collaborations in Health Services Research: A Case Study from Qatar

**DOI:** 10.5539/gjhs.v4n6p148

**Published:** 2012-09-25

**Authors:** Maya M. Hammoud, Maha Elnashar, Huda Abdelrahim, Amal Khidir, Heather A.K. Elliott, Amal Killawi, Aasim I. Padela, Abdul Latif Al Khal, Abdulbari Bener, Michael D. Fetters

**Affiliations:** 1 Department of Obstetrics and Gynecology, University of Michigan Medical School, United States; 2 The Center for Cultural Competence in Healthcare, Weill Cornell Medical College in Qatar, Qatar; 3 Department of Pediatrics, Weill Cornell Medical College in Qatar, Qatar; 4 School of Public Health, University of Michigan, United States; 5 School of Social Work, University of Michigan, United States; 6 Program on Medicine & Religion/Faculty, University of Chicago, United States; 7 Department of Medical Education, Hamad Medical Corporation, Qatar; 8 Department of Medical Statistics and Epidemiology, Hamad Medical Corporation, Qatar; 9 Department of Family Medicine, University of Michigan Medical School, United States

**Keywords:** international collaborations, global partnerships, international research, health care quality, cultural adaptations

## Abstract

Economic globalization and advances in technology have made it more feasible and even necessary to develop international research collaborations in global public health. Historically, collaborations in global research described in the literature have been mostly “North-South” collaborations in which the more developed “North” country works together with a developing “South” country to conduct research in the latter. This type of collaboration has for the most part, represented unequal partnership and rarely left behind a lasting impact. Recently, the opportunity for a new kind of international research partnership has emerged in which the host country has significant financial resources, but relatively limited expertise in research methodology or techniques and research implementation. This type of collaboration features a relative equalization of power between the international partners. The purpose of this paper is to describe the process of building a successful research collaboration between a team in the United States and a team in Qatar, a rich Arabic nation in Gulf. We present a case study that provides an overview of our own project focused on the development of a culturally and linguistically adapted health care quality instrument for Qatar, discussing many of the benefits and challenges we encountered during each phase of instrument development. We present recommendations for researchers seeking sustainable and equitable partnerships with the Arab World.

## 1. Introduction

Economic globalization and advances in technology have made it more feasible and even necessary, to develop international research collaborations in global public health. Historically, collaborations in global research described in the literature have been mostly “North-South” collaborations in which the more developed, traditionally high income, “North” country works together with a developing, middle or low income, “South” country to conduct research in the latter. This type of collaboration has for the most part, represented unequal partnership and rarely left behind a lasting impact. Once the work is complete, there is usually no research infrastructure left behind in the developing country (Glickman et al., 2009).

Recently, the opportunity for a new kind of international research partnership has emerged in which the host “South” country is the high income country with significant financial resources, but relatively limited expertise in research methodology or techniques and research implementation. This type of collaboration features a relative equalization of power between the international partners. One from a position of research experience and expertise and another possess in global cultural expertise, manpower and financial support needed to develop a research infrastructure. Such is the case in the State of Qatar, one of the fastest growing scientific and economic countries in the world.

Qatar’s economy began transforming rapidly with discovery of the world’s third-largest natural gas field (U.S. Department of State, 2012). The country’s leadership resolved to invest heavily in developing a science city in the capital Doha, that features collaborative educational affiliations with leading institutions around the world in strategically important fields. In the medical field, Qatar has been making major advances in the quality of healthcare (Bener & Al Mazroei, 2010) and established the Weill Cornell Medical College-Qatar (WCMC-Q) and the Sidra Medical and Research Center for biomedical research. These two institutions lead the country’s efforts in the advancement and promotion of medical education and research. In order to further promote research endeavors and collaboration, Qatar also established the Qatar National Research Fund (QNRF) in 2006 with the aim to develop Qatar as a knowledge-based economy through funding original research and parallel building of research infrastructure (QNRF, 2012). The National Priorities Research Program (NPRP) is the flagship program of QNRF and in many ways emulates the NIH Research Project Grant RO1 in the United States. Our research group applied and received an NPRP grant in 2009.

It has been well acknowledged that global public health research collaborations require a complementary set of competencies because of the additional knowledge and skills needed to succeed in a global environment (Cole et al., 2011). For example, skills in cross-cultural communication and the willingness to be mentored and mentor others are essential. Yeatts and colleagues (2012) described challenges faced when researchers from the Gillings School of Public Health at the University of North Carolina conducted environmental health research in the United Arab Emirates. They also emphasized acknowledging cultural differences and the importance of flexibility and careful planning. The purpose of this paper is to describe the process of building a research collaboration with Qatar, an Arabic nation in Gulf. We present a case study that provides an overview of our own project focused on the development of a culturally and linguistically adapted health care quality instrument for Qatar, including many of the benefits and challenges we encountered during each phase of instrument development. Recommendations for researchers seeking sustainable and equitable partnerships with the Arab World will be discussed.

## 2. Case Study

### 2.1 Setting

Considerable progress has been made over the past ten years in the provision of quality health care in Qatar. In 2007, the Joint Commission International (JCI) accredited Hamad Medical Corporation (HMC), the main and largest public health care provider in Qatar. With full support of Qatar’s leadership and the accreditation’s required standards, HMC is committed to upgrading health care delivery systems and ensuring access to high quality health care for the entire Qatari population, including a very large and extremely diverse expatriate population of various ethnicities and nationalities. As research elsewhere has demonstrated, diverse national populations often have disparities in access to care as well as in healthcare utilization. This in turn contributes to poorer health outcomes among minorities and one would expect Qatar to be no exception (President’s Advisory Commission on Consumer Protection and Quality in the Health Care Industry, 1998; Office of Minority Health, 2000; National Institutes of Health, 2002). However, research aimed at bettering understanding the diverse health needs and outcomes of Qatar’s population specifically and the Arab population generally is scant, and as a first-step Qatar’s health care providers require validated health care quality assessment tools. Thus, our project’s main aim was to develop a culturally and linguistically adapted health care instrument that can be used to assess the quality of health care in Qatar.

### 2.2 Developing an Initial Research Team

At the time of project initiation in 2009, Qatar had a limited biomedical research infrastructure through Weill Cornell Medical College in Qatar (WCMC-Q), and Hamad Medical Corporation (HMC), the largest hospital the designated teaching institution for the medical school. A local faculty member at WCMC-Q conceived the project and contacted a colleague at the University of Michigan Medical School (UMMS) in the United States known for his expertise in cross-cultural research. Together, they assembled a multi-disciplinary international team with 17 members from Qatar and the United States. The team developed and submitted an application for funding in the second round of requests for applications from QNRF. This project was funded after the first submission.

The mutual process of building a team of collaborators from both Qatar and the US began during the preparatory phase to support writing the grant proposal. Their involvement was critical in designing the methods, assessing the local culture, navigating through the local politics for formal and informal authorizations to conduct the project and for planning eventual dissemination. All members played crucial roles contributing to the success of the project and collaborative efforts. The Qatar-based team included faculty from WCMC-Q and staff from its Center for Cultural Competence in Health-Care (CCCHC) (Elnashar et al., 2012) and faculty from HMC. The CCCHC had already been working closely with HMC on establishing interpreter services and training within the hospital. This solidified them as a natural partner in WCMC-Q given their existing connections with HMC. Within HMC, the main partners included the Chairman for Internal Medicine, who in addition to being interested in providing high quality care, could facilitate a trusting relationship between the research team, HMC departments, and the HMC Research Center. He would also assure that the project activities met HMC policies and regulations. In addition, the team recruited the main biostatistician from HMC for expertise on local data collection procedures and for the data analysis phase of the project. This networking through personal relationships proved very helpful for implementation of the project.

The project team skills from members in the US complemented the team in Qatar. In addition to the lead co-principal investigator who brought experience in conducting cross-cultural research and mixed-methods studies, additional investigators with knowledge of Arab and Muslim culture were recruited. A US biostatistician and qualitative data analyst were added to the team for the analysis phase of the project.

### 2.3 Selection of a Quality Assessment Instrument for Adaptation

The team members considered the option of developing a new instrument or using an existing instrument and socio-culturally adapting it to Qatar. After considerable discussion, the team opted to adapt an instrument, as this could facilitate international comparison in spite of socio-cultural adaptations. The United States Agency for Healthcare Research and Quality (Agency for Healthcare Research and Quality [AHRQ], 2010) has heavily invested in and supported the Consumer Assessment of Healthcare Providers and Systems (CAHPS), a multi-year program designed to support and promote the assessment of consumers’ experiences with health care (AHRQ, 2008). This public-private initiative was developed in an effort to standardize surveys of consumers’ experience with health care, as well as in response to patient demand for data regarding the quality of care delivered by different health plans (Collins et al., 2002; Davis et al., 2002; National Committee for Quality Assurance, 2003a; National Committee for Quality Assurance, 2003b). Adapting the CAHPS program using standardized patient questionnaires is useful in a variety of health care settings. The team and future researchers would be able to compare results of individual providers and institutions over time. With funding support from the Qatar National Research Fund (QNRF), the multidisciplinary research group set out to develop a culturally and linguistically adapted CAHPS Health Plan Survey fondly called the CAHPS-Q for the country of Qatar in the four predominant languages spoken in Qatar: Arabic, English, Hindi, and Urdu.

### 2.4 Implementation

Following approval from QNRF, the team set out to implement the project. Table 1 depicts an overall timeline of the project at the time of the writing of this paper. The table provides a comparison between the anticipated study timeline vs. how long the process actually took up to Phase II. Please note that Phases III, IV and V are either ongoing or have not yet begun. Although it is usual for international major projects to take longer, this project faced delays in its initial phases more than anticipated. Below, we address obstacles related to the collaborative nature of the work that caused timeline delays and discuss strategies used to overcome these obstacles.

**Table 1:** A comparison of the proposed vs. actual study timeframe through Phase II

	Proposed Timeframe	Actual Completion	Comments
**Phase I**	6 months (Months 0-6)	10 months (Months 0-10)	UMMS IRB approval of this phase took 6 days. The short timeframe was due to lack of human subject involvement in the instrument translation phase.
			WCMCQ - Phases 1-3 were exempted as the study team was not collecting patient information
			HMC IRB approval of this phase took 32 days.
Hire Remaining Staff	Within 6 months	Month 5	Staff hires took 3 months more than expected.
Translate Instruments	Within 6 months	Month 9	Translations completed in number of months expected.
Develop Data Collection	Within 6 months	Month 8	Delay of 2 months due to time needed to hire qualified staff.
Interview Guide Demographics		Month 10	Instrument development delay due to complexity of assessing cultural and educational background.
**Phase II**	12 months (Months 7-18)	22 months (Months 11-33)	Delay occurreddue to need for developing a new acculturation instrument, the MAI-Q, appropriate for the setting.
			HMC approval took between 1 to 4 days for all parts of this phase.
Phase IIa			UMMS IRB approval of this phase took 41 days.
Recruitment	Within 6 months	Month 18	
Data Collection	Within 12 months		Delay due to difficulty identifying qualified Arabic staff and turnover due to higher paying jobs.
Urdu, Hindi and English		Month 22	
Arabic		Month 29	
Phase IIb – MAI-Q Cognitive Testing	Added	Month 31	US IRB approval of this phase took 38 days.
Phase IIc – MAI-Q Final	Added	Month 33*	US IRB approval of this phase took 47 days.
**Phase III**	5 Months (Months 19-24)	5 Months* (Months 33-38)	Anticipated concerns: Not being about to do cognitive testing outside of the hospital in Qatar. Due to unavoidable subject time restraints Cognitive testing of the CAHPS-Q will have to be broken down into sections. The challenge that this will cause is the study team will have to do a lot more CAHPS-Q cognitive interviews than anticipated.
			UMMS IRB approval of this phase took 58 days.
			HMC IRB approval of this phase took 3 days.
**Phase IV**	5 Months (Months 25-30)	4 Months* (Months 39-43)	
**Phase V**	6 Months (Months 31-36)	6 Months* (Months 44-50)	

* Indicating the phase is ongoing or not yet started

### 2.5 Obtaining the Approval of Institutional Review Boards

Since the project included investigators from the UMMS, WCMC-Q, and HMC, the team needed institutional review board (IRB) approval from all three institutions. Approvals of the three institutions occurred in various ways at different phases in the study. The IRB approval at UMMS for phase I was relatively quick as it only involved translating existing documents, however, approval for subsequent phases including the process of amendment submission, revision, and resubmission took substantively longer, 38 to 58 days. At WCMC-Q, Phases I to III were deemed exempt based on the assessment that these phases were low risk and only involved interview procedures, and the approval was quickly obtained. A different story unfolded with the HMC IRB. Phase I approval took a total of 32 days, even though it only involved translation of documents. We attributed this in part to the fact the project was completely new to the reviewers and possibly due to the fact that the IRB was relatively new and still in the process of developing standard procedures. Additionally, confusion occurred regarding the role of the IRB in the research enterprise. Recommendations for changes in the research protocol itself were requested that included expanding the project to incorporate additional patient populations. This was resolved after explaining the rationale for the population choices and the limitations due to the project funding. However, subsequent approvals occurred quickly, within1 to 4 days. We attributed this excellent turnaround to clarification of the IRB’s role to human subject protection concerns, continuity of the IRB’s intimate knowledge of the project, and perhaps a smaller volume of projects that allowed attentiveness to our project. The need to submit amendments to the IRB for each phase of instrument development continues to be a time-consuming and laborious process.

### 2.6 Establishing a Full Research Team

The recruitment and training of the local field team was integral in assuring proper data collection and storage, maintaining patient confidentiality, and overall success of the research project. After the core investigators and study personnel were assembled and IRB applications were approved, local field interviewers were hired (Table Given the high degree of linguistic and cultural heterogeneity in Qatar population, several factors had to be considered in the hiring and training of the interview staff. These included the following:

1) Gender of the staff. Qatar is a conservative Muslim Arabic country where male and female services are delivered in separate locations. Thus, it is not acceptable for a man to enter the ‘women’s only’ area, even for research purposes only. However, the reverse does not necessarily hold true. A female research assistant may enter the male only areas. Therefore, all research staff hired were females.
2) Diversity of the research staff. Since the project included administering a survey in multiple languages, it was necessary to recruit women of different nationalities who were fluent in these languages. This presented some difficulties as it was challenging to find women with relevant skills or background who could be trained for the project. Women expatriates looking for work in Qatar are typically trailing spouses who are in Qatar due to husbands’ jobs. They cannot be recruited directly from their home countries due to the grade level of these positions which would not support relocation or benefits inside Qatar.
3) Training of the research staff. Training had to occur in multiple phases. The US Lead Co-PI and Co-Investigators, as well as the core investigational team in Qatar, trained the research staff in conducting interviews, transcribing recordings, and translating the Urdu, Hindi, and Arabic transcriptions into English. The training consisted of a two-day workshop held in Qatar and included instruction in interviewing skills, confidentiality, and ethics training. The workshop included sessions with role-playing and mock interviews. This instruction was provided to ensure that the field interview team was well trained in conducting interviews and gathering data in the field, as well as to create a knowledge infrastructure that the staff could rely on to provide training to future research staff. Research assistants also conducted trial interviews with their family members, colleagues, and friends to calculate the approximate interview duration and to assess the clarity and comprehension of the questions.

### 2.7 Establishing Communication Methods

Successful international project implementation requires constant and flexible communication between the research teams. At the initiation of the project, all investigators agreed on regularly scheduled meetings with their local teams as well as between the two international research teams as follows:

- Weekly meetings in the US for the US team
- Weekly meetings in Qatar for the Qatar team
- Weekly Skype® meetings between the US and Qatar team which also included screen sharing using Adobe Connect®.

In addition to the regularly scheduled meetings, the following methods of communication were used:

1) Email: An email group was established for the entire research team. Any member of the research team can initiate an email to the rest of the team.
2) Telephone conferences: Scheduled as needed between various team members.
3) Site visits: Site visits were conducted once a year by members from the US team to Qatar. In addition, two senior members from the Qatar team visited the US for training about project methodology.

Most importantly, all team communication and documents were uploaded in a virtual online collaborative space on a secure server for access by all team members.

## 3. Case Analysis

Cross-cultural and international collaborations face many challenges and have many benefits associated with project development and execution. Many of the challenges include cultural, institutional, and geographic differences discovered as the project members execute the project, and these can cause time delays. The discussion below presents many of the challenges that the US and Qatar teams encountered, as well as many of the benefits accrued as a result of this collaborative process.

### 3.1 Collaboration Challenges

In any international collaboration, a wide variety of challenges will arise. This section does not provide an exhaustive list of the challenges that were encountered, but instead highlights the issues that provided constant and/or significant limitations to the research process and collaborative environment. Many of these involved geographic and cultural limitations.

#### 3.1.1 Managing Differences in Work Schedule

Differences in work schedules manifested in a variety of ways each presenting its own challenge to the efficient progress of the project.

1. Time zone differences. The difference in time zones between the two countries made it challenging to schedule meetings when all project members were available. Because our project is dependent on knowledge integration between and across all team members, this challenge was especially noteworthy. Furthermore, Qatar does not follow daylight savings, which further increases the time zone difference for half the year.
2. Work hours. The workweek in Qatar is from Sunday to Thursday while in the US, it is Monday to Friday; this effectively shortens the collaborative workweek to 4 days. In addition, during the summer months, some institutions have reduced work hours and many employees take extended vacations to escape the heat. This also reduces the work hours as well the efficiency by which certain processes are completed.
3. Holiday schedules. Qatar, as an Islamic country, has many major holidays that do not coincide with the holidays in the US. In addition, during the month of Ramadan, the work hours are significantly shortened in certain institutions, further affecting the speed of processing regulatory issues such as with an IRB or the speed of recruiting patients. Therefore, it is important to plan way ahead of time around certain cultural or religious holidays, which can be quite different and extend over longer periods of time.

#### 3.1.2 Varying Cultural Backgrounds

The differences in cultural background present multiple challenges to the execution of the project.

1. Difference in communication styles. Cultures have different communication styles. For example, communication in the Arab culture is not always direct, tends to be less confrontational and spoken politely. In this project, interpretation beyond what is being said, i.e., “interpreting between the lines”, had to occur at times, and this was especially challenging where most of the communication occurred over the phone or email where one could not obtain any cues from non-verbal communication. Therefore, face-to-face meetings during the international visits became extremely important to resolve conflicts or misinterpretations.
2. Subject recruitment. Finding and recruiting participants required more time investment than what the US collaborators were accustomed to due to different expectations. Subjects in Qatar cannot be recruited simply by the usual advertising methods used in the western world. Regular mail, although existent, is not commonly utilized by the general population. Most individuals approached did not know their home address and so procedures requiring follow-up were different from what the researchers expected. Research assistants needed to directly interact with eligible subjects and provided detailed explanations about the project procedures and assurances about privacy. However, many subjects showed reluctance to entrust their private information with the research assistants. Although legislators in Qatar have passed well-developed patient privacy laws, as well as the presence of ethical norms in research, these were unknown to many eligible subjects. In Qatar, research investigations remain relatively few, so the population is not fully aware of legislation. In addition, some cultural variations such as having separate entrances for men and women patients in the clinic also required additional time investment.

#### 3.1.3 Retention of Research Staff

Qatar is a small wealthy country where the majority of the population are expatriates who work on time-limited assignments. The local Co-Lead PI had to screen candidates carefully to identify candidates intending to be in Qatar for the duration of the project to ensure continuity. Following initial training, research assistants become more attractive to other local investigators for recruitment into their projects and they are offered better financial incentives as the supply of trained research staff is limited. Retention was best accomplished by creating a desirable team environment for the research staff. Somewhat ironically, the greatest challenge involved finding, hiring, and retaining Arabic speaking employees, even though Qatar is an Arabic speaking country. The team learned about the competitive market and demand for Arabic speaking employees who can easily find other better paying job opportunities. Once again, the team environment proved to be a key factor in maintaining the Arab individuals who made up part of the research staff.

#### 3.1.4 Developing Survey Instruments

Even though the survey instrument was being adapted from an existing survey, socio-cultural adaptations presented many challenges.

1. Defining demographic backgrounds relative to birthplace, heritage culture, and nationality. Describing the demographics of a diverse population was quite arduous. It took many hours of explanation in order for the US team to understand the issues surrounding residency and citizenship in Qatar. Qatar’s population uniquely consists mostly of expatriates who function primarily as laborers in the booming economy. When team members attempted to define certain demographic categories, it became quite clear the typical demographic dimensions used in the United States do not apply. For example, being a resident in Qatar does not hold the same meaning as being a resident in the United States. Expatriates are typically given time-limited working permits, and they can never become citizens of Qatar. In addition, a large number of people hold multiple citizenships and speak multiple languages, an issue rendering it difficult to define their main citizenship or language. Table 2 illustrates the subjects’ backgrounds by country of birth, ancestry, and nationality on their residence permit and demonstrates the diversity of enrolled subjects. Table 3 depicts specific individuals who reported differences in their birth, ancestry and/or nationality, and further demonstrates the extent of variation within an individual subjects’ background. Together, these two tables illustrate the challenges of describing demographic features of the population for purposes of understanding who participated in the research.

**Table 2:** Subject background by country of birth, ancestry, and nationality on resident permit

Region	Country of Birth		Area of Ancestors		Nationality on Residence Permit	
	Count	Percent	Count	Percent	Count	Percent
**Northern Europe**	**10**	**12**	**11**	**13**	**8**	**10**
United Kingdom	2		4		0	
British	1		0		8	
Scotland	1		0		8	
England	3		1		0	
Norway	4		5		0	
**Northern America**	**0**	**0**	**0**	**0**	**1**	**1.2**
America	0		0		1	
**Northern Africa**	**7**	**8.3**	**8**	**9.5**	**8**	**9.5**
Egypt	6		6		6	
Tunisia	1		2		2	
**Eastern Europe**	**1**	**1.2**	**1**	**1.2**	**1**	**1.2**
Romania	1		1		1	
**South-Eastern Asia**	**5**	**6**	**4**	**5**	**5**	**6**
Philippines	5		4		5	
**Southern Europe**	**0**	**0**	**1**	**1.2**	**1**	**1.2**
Italy	0		0		1	
Spain	0		1		0	
**Southern Africa**	**1**	**1.2**	**0**	**0**	**0**	**0**
South Africa	1		0		0	
**Southern Asia**	**40**	**48**	**41**	**49**	**41**	**49**
India	14		17		14	
Nepal	3		3		3	
Pakistan	20		18		22	
Bangladesh	1		1		1	
Sri Lanka	1		1		1	
Persia	1		0		0	
Afghanistan	0		1		0	
**Western Europe**	**1**	**1.2**	**1**	**1.2**	**1**	**1.2**
Belgium	1		1		1	
**Western Africa**	**1**	**1.2**	**1**	**1.2**	**1**	**1.2**
Nigeria	1		1		1	
**Western Asia**	**16**	**19**	**15**	**18**	**15**	**18**
Qatar	7		5		8	
Palestine	5		6		3	
Syria	1		1		0	
Yemen	1		2		0	
Jordan	1		0		3	
Bahrain	1		1		0	
Lebanon	0		0		1	
**Central Asia**	**1**	**1.2**	**1**	**1.2**	**1**	**1.2**
Kazakhstan	1		1		1	
**Australia and New Zealand**	**0**	**0**	**0**	**0**	**1**	**1.2**
Australia	0		0		1	
**No Response**	1	1.2	0	0	0	0
	84	100	84	100	84	100

**Table 3:** Variation in individual subject’ background by country of birth, ancestry, and nationality on resident permit among individuals with diverse backgrounds

	Country of Birth	Area of Ancestors	Nationality on Residence Permit
**1**	Philippines	Spain	Philippines
**2**	Scotland	Scotland and Norway	British
**3**	Scotland	UK	American
**4**	Qatar	Afghanistan	Pakistan
**5**	Syria	Syria	Jordanian
**6**	Scotland	UK	British
**7**	South Africa	UK	Italian
**8**	Kazakhstan	Kyrgyzstan	Kazakhstani
**9**	England	England	Australia
**10**	Qatar	Pakistan	Pakistani
**11**	Palestine	Palestine	Lebanon
**12**	**	Yemen	Qatari
**13**	Pakistan	India	Pakistani
**14**	Pakistan	India	Pakistani
**15**	Pakistan	India	Pakistani
**16**	Qatar	Tunisia	Tunisian
**17**	Yemen	Yemen	Qatari
**18**	Palestine	Palestine	Jordanian
**19**	Jordan	Palestine	Jordanian
**20**	Bahrain	Bahrain	Qatari
**21**	Persia	Qatar	Qatari

** missing
2. Educational systems. The level of participants’ education also emerged as another surprisingly challenging demographic element requiring careful consideration. While the host country collaborators raised this issue early on, a systematic search of the educational systems of the various countries demonstrated the particular difficulty in even attempting to quantify years of education. It was quite challenging for the Qatar team to fully research and develop the appropriate categories for describing the educational systems of the extremely diverse target populations. As illustrated in Table 4, participants from the eligible population in Qatar could have studies in over 20 different educational systems. Since many subjects are illiterate, researchers working in the region must consider carefully how to measure educational background with reference to the goals of the research. Future researchers might want to consider building in additional time to simply define demographic elements as it may require an extended period of time to research and summarize the data in a useful format.

**Table 4:** School systems in the region

Country	Primary School		Middle School		High School		Certificate/credential obtained

	Name of stage	Number of Years	Name of stage	Number of years	Name of stage	Number of years	
Algeria	Primary	Nine years	-----------	-----------	Secondary	Three years	Baccalauréat^$^
Egypt	Primary	Six years	Preparatory	Three years	Secondary	Three years	ThanaweyaAama^&^
India	Primary	Four years (Excluding 2 years of kindergarten)	---------	---------	Secondary	Six Years	S.S.C.- Secondary School Certificate
Iran	Primary	Five years (age 6 -11)	Middle (Guidance)	Three years (age 11 to 13)	Secondary	Four years (age 14- 17)	
Iraq							
Jordan	Primary	Six years	Preparatory	Three years	Secondary	Three years	ThanaweyaAama
Kuwait	Primary	Four years	Elementary	Four years	Secondary	Four years	ThanaweyaAama
Lebanon	Elementary	Six years	Intermediate	Three years	Secondary	Three years	Baccalaureate
Libya	Primary	Nine years			Secondary	Three years	
Morocco	Primary	Six years	Lower-middle/intermediate school	Three years	Upper secondary	Three years	Baccalaureate
Oman	Primary	Six years	Preparatory	Three years	Secondary	Three years	Thanaweya Aama
Pakistan	Primary	5 years	Middle	Three years	Secondary	Two years	
Palestine (Ghaza)	Primary	Three years	Preparatory	Three years	Secondary	Three years	Thanaweya Aama
Qatar	Primary	Three years	Preparatory	Three years	Secondary	Three years	Thanaweya Aama
Saudi Arabia	Primary	Three years	Preparatory	Three years	Secondary	Three years	Thanaweya Aama
Sudan	Primary	Eight years			Secondary	Three years	Thanaweya Aama
Syria	Primary	Six years	Lower Secondary	Three years	Upper Secondary	Three years	Baccalaureate
Tunisia	Primary (Premier Cycle)	Six years	Primary (Second Cycle)	Three years	Upper Secondary	Four years	Baccalaureate
U.A.E.	Primary	Six years	Preparatory	Three years	Secondary	Three years	Secondary school certificate
Yemen	Primary	Nine years	*Certificate: Intermediate school certificate	Upper secondary	Three years	Thanaweya Aama	

$ Baccalaureat (French for baccalaureate)

& ThanaweyaAama (Arabic for general baccalaureate)
3. Cultural adaptation. The cultural and linguistic adaptation of the research instrument involved a very long and arduous process. The US and Qatar teams spent many hours discussing the nuances of language adaptation by identifying the best words and phrases for use in the survey instrument. Some words and phrases cannot be translated literally as the need to account for the sociocultural context emerges as relevant. For example, the key concept of a primary care physician does not exist widely in Qatar. In translating, consideration had to be given to the understanding of a personal or a general doctor. In another example, the words “specialist” and “consultant” are used to represent the seniority of the attending physicians rather than the particular expertise of the individual or the role of the physician as in the US. Fleshing out the cultural and social understanding of fundamental terms is absolutely critical to ensuring research objectives are met and cultural accuracy and sensitivity are maintained.

All of these challenges required significant effort and time to reconcile, which at times slowed down the progress and compromised the timeline of the project. The investigators estimate the project was delayed about 10 months due to the geographic, cultural, and linguistic differences. Any researcher who wishes to engage in international collaborations in the Arab world needs to be aware that these challenges exist. Therefore, it is important to ensure that there is time built into the research project to address these issues.

### 3.2 Collaboration Benefits

While the project presents many challenges to the researchers involved, the development of a culturally and linguistically adapted health care survey instrument remains very important. Collaborating with international colleagues is crucial to developing a cultural informed instrument that can subsequently be implemented in Qatar. International collaboration strengthens the project and benefits the researchers because it:

1. Offers a more holistic and global approach to the issues being investigated. Team members came from many different cultures and backgrounds, which contributed to the richness and culturally informed nature of the discussions.
2. Provides multiple and different sets of resources and skills to help facilitate research that otherwise would not have been conducted. For example, the US team provided expertise in research methodology and principles in approaching cultural influences on decision- making, and the Qatar team provided cultural expertise, adaptation of methodology to suite the Qatar context, funding, and the access to a special and diverse patient population.
3. Creates a network of researchers and provides collaborators with opportunities to gain knowledge from other research systems, and helps to build strategic relationships that could work to develop further research in their areas of interest.
4. Helps disseminate information in the field at a faster rate globally. Both teams will be disseminating their research findings in their respective geographical regions which are thousands of miles apart. Provides multiple benefits to each country. For example, at the end of this project, Qatar will possess a validated instrument to study and improve the quality of patient care for many socially and linguistically diverse groups in Qatar. The US team will have benefited from working and learning with a multicultural and diverse group of researchers.

With growing degrees of globalization, these collaborations are even more important, especially in Arab countries that have been shown to lag behind other Middle Eastern countries in biomedical research (Benamer & Bakoush, 2009). Specific needs have been identified in certain research arenas such as congenital heart disease (Farhat et al., 2012) and mental health (Osman & Afifi, 2010). These partnerships open cultural doors that lead to better understanding of diversity and contribute to improvements in health care locally and globally.

## 4. Recommendations

The collaboration on this 3-year project provided the researchers on both teams with insights into the keys to success (Table 5) and the steps required to develop a fruitful and ongoing partnership. Additional competencies pertaining to the global context and cultural differences are needed to succeed in global partnerships (Cole et al., 2011). While our study is limited by being confined to a collaboration with one country and one project, knowledge of the challenges, as well as the benefits, of such a relationship will be crucial to researchers looking to develop and form global public health research teams. The steps to a successful partnership include:

**Table 5:** Keys to success in international collaborations with the Arab World

Key to Success	Importance
Personal connections	Provides a conduit to establish teams which are trusting of one another
High level buy-in	Offers the support needed to conduct the research and implement results
Cultural competence	Assures understanding of local customs and habits and preserves mutual respect
Patience	Prevents development of frustrations
Flexibility in communication methods	Assures continues and frequent contact which might not be feasible otherwise

1. Defining the purpose of the collaboration: All collaborators involved in the project need to understand the importance of the project to both sides and expectations need to be set early in the project.
2. Building a mutually beneficial and trusting partnership: In addition to finding the right partners with their respective areas of expertise, support is required at the highest level of the organization, and trust at all levels.
3. Investing in professional education: Professional development needs to be planned and accounted for in the budget; international collaborators need to learn about the local culture and vice versa. Mutual respect must to be applied.
4. Developing a sustainable infrastructure: Developing local champions with their specific areas of expertise is important in assuring the success of the project and in building long term sustainable intellectual capacity.
5. Accounting for local culture: Researchers must understand the local culture and involve local experts in the process in order to be effective. This will also help in translating research outcomes into practice.

Despite all the challenges the study team has encountered, every individual on the team has remained very committed to the project and we have been able to reach homeostasis in research collaboration. The instrument developed is being delivered and the research team is collaborating on writing and disseminating the study outcomes.

## 5. Summary

Collaborations with the Arab World where research expertise is primarily provided by a partnering country and the financial support primarily provided by the wealthy Arab country has rarely been described in the literature. Our project describes emphasizes building a research infrastructure in anticipation of local researchers becoming independent in the future.

While it might have been simpler to utilize the host country’s financial support and invest little time and effort into developing its research infrastructure, our project ensured that expertise was utilized from both teams and that appropriate training for Qatar research staff occurred. Both teams will retain full access to the data collected during this study, ensuring that data is available for future analysis. The US team worked to provide the appropriate mentoring and direction to the local team while maintaining cultural sensitivity. Additionally, the Qatar team provided excellent cultural and linguistic knowledge and was indispensible in facilitating the creation of the survey instrument and subject recruitment. We hope this report will help to facilitate smooth transitions and globalization in international health services research.
